# A Comparative Analysis of Three Antioxidants in Addition to Scaling and Root Planing in Stage Three Grade B Periodontitis

**DOI:** 10.7759/cureus.51916

**Published:** 2024-01-08

**Authors:** Sagorika Saha, Sonal Mahilkar, Dennis V Abraham, Sreejith S, Nagesh Bhat, Dr Shilpi Srivastava

**Affiliations:** 1 Department of Periodontology, Chhattisgarh Dental College and Research Institute, Rajnandgaon, IND; 2 Department of Dentistry, Government Medical College, Mahasamund, IND; 3 Department of Periodontics, Maitri College of Dentistry and Research Center, Durg, IND; 4 Department of Oral Pathology and Microbiology, Dent Inn Dental Clinic, Thiruvananthapuram, IND; 5 Department of Preventive Dental Sciences, Al Baha University, Al Baha, SAU; 6 Department of Oral Medicine and Radiology, Teerthanker Mahaveer Dental College, Moradabad, IND

**Keywords:** stage iii periodontitis, lycopene, septilin, green coffee bean extracts, antioxidants

## Abstract

Introduction: The main feature of periodontitis is the development of periodontal pockets as a secondary consequence, which is mainly caused by an excessive immune response to the dental biofilm. The prime factor in the pathogenesis of periodontitis is an increase in oxidative stress. Numerous clinical studies have demonstrated that antioxidant supplementation can reduce endogenous antioxidant depletion and the oxidative damage that goes along with it. Hence, antioxidant therapy in the treatment of periodontal disease may prove to be a promising tool.

Objective: The objective of the study is to compare the efficiency of three different antioxidants as oral supplements.

Materials and methods: Eighty patients with chronic periodontitis were randomly split into four groups. The control group received scaling and root planning (SRP), and the test group received oral supplements for 30 days with SRP. Pocket depth (PD), clinical attachment level (CAL), oral hygiene index-simplified (OHI-S), and sulcus bleeding index (SBI) were evaluated at baseline and 30 days. The analysis of the data was done with ANOVA, Kruskal-Wallis, and post hoc tests. The significance level was set at p<0.05 and p<0.001.

Results: All groups resulted in a statistically significant reduction in all parameters from baseline to one month. The treatment groups revealed a statistically significant reduction in PD and CAL (p<0.00) but no reduction in OHI-S and SBI (p>0.05) scores. A highly statistically significant reduction was observed in PD with green coffee bean extract when compared with other groups.

Conclusion: Green coffee bean extract oral supplements may prove to be a promising appendage in therapeutic and prophylactic fashion, along with SRP, in the treatment of stage III grade B periodontitis patients.

## Introduction

Periodontal disease (PDD) is an inflammatory state characterized by continuous loss of attachment apparatus, leading to the loss of teeth. There are multiple factors responsible for periodontal disease, including periodontal pathogens and oxidative stress [1]. Pathogens causing PDD are *Porphyromonas gingivalis *(*P. gingivalis*), *Fusobacterium nucleatum* (*F. nucleatum*), *Aggregatibacter actinomycetemcomitans* (*A. actinomycetemcomitans*), *Prevotella intermedia* (*P. intermedia*), etc. An immune response is initiated as a pathogen habitat in the local periodontal tissues by fibroblasts and macrophages by the production of cytokines such as factor‑α (TNF-α) and interleukins (IL) 1 and 6 as mediators of the inflammatory response and immune reaction [2].

A recent definition of oxidative stress is "an imbalance favoring oxidants over antioxidants, resulting in a disruption of redox signaling and control and/or molecular damage" [3]. The oxidation of a substrate can be greatly delayed or inhibited by chemicals called antioxidants, which are present in lower concentrations than those of an oxidizable substrate [4]. The antioxidant defense system has the ability to mitigate the adverse effects of free radicals and other non-radical reactive species [5]. The body's organic antioxidant security system is overpowered by reactive oxygen species (ROS) during an inflammatory reaction, resulting in oxidative stress. The primary cause of the pathogenesis of periodontitis is an increase in oxidative stress. Hence, antioxidant therapy can improve periodontal health [1].

There are various antioxidants available on the market as oral supplements. In the present study, we have compared three antioxidant oral supplements: chlorogenic acid (CGA), lycopene, and Septilin, for the treatment of stage III, grade B periodontitis. Chlorogenic acid is a naturally generated polyphenolic compound. Chlorogenic acid is structurally composed of an acid-caffeic ester with three quinic acid hydroxyl groups [6] and is synthesized by esterification of cinnamic acid. The medical applications of CGA include but are not limited to, antibacterial [5], antifungal, antiphlogistic, antioxidant, antiviral, and chemopreventive activities. One of the primary organic forms of CGA is green (or raw) coffee (5-12 g/100 g) [3]. Lycopene is a compound of the beta (β)-carotenoids family [5]. It is an open-chain isomer of β-carotene and functions as a natural antioxidant with a high quenching rate [1]. The scarlet hue of produce and fruits is due to a natural pigment that is synthesized by lycopene. The biological impacts of lycopene are linked to its non-oxidizing and antioxidant properties, which include cell function in signaling and anti-inflammatory properties [4].

A herbal remedy called Septilin (Himalaya Wellness Company, Makali, Bengaluru) is reported to be antimicrobial, anti-inflammatory, reduce oxidative stress, and have wound-healing characteristics. It is also claimed to help treat infections from both gram-positive and gram-negative bacteria. The mechanism explains how Septilin governs the production of proinflammatory mediators in lipopolysaccharide-stimulated macrophage and monocyte cells, which involves IL-6, IL-8, TNF-α, nitric oxide, cycloxygenase (COX-2), and phosphodiesterase [4]. Septilin is found in Blasmodendron mukul and Shankha Bhasma powders, in addition to extracts from six different plants [7].

To the best of our knowledge, no studies have been done to date on comparing the three antioxidants in stage III grade B periodontitis.

## Materials and methods

Inclusion criteria

The inclusion criteria were as follows: 1. systemically healthy patients; 2. pocket depth (PD) ≥6mm; 3. clinical attachment level (CAL) ≥5mm; 4. stage III periodontitis; 5. grade B periodontitis

Exclusion criteria

The exclusion criteria were as follows: 1. history of antibiotic use in the previous three months; 2. individuals with a history of periodontal disease treatment within six months; 3. pregnant or lactating women; 4. smokers and alcoholics; 5. patients who require antibiotic prophylaxis

A total of 80 chronic periodontitis patients were randomly split into four groups, with 20 patients in each group. The control group, i.e., Group A, received only scaling and root planning (SRP). Group B received lycopene 395 mg oral supplement adjunct to SRP; Group C received Septilin 0.07kg oral supplement adjunct to SRP; and Group D received green coffee beans extract 800mg per serving oral supplement adjunct to SRP. The ethics committee of Maitri College of Dentistry and Research Centre, Durg, India, approved the study with the ethical committee clearance certificate reference number MCDRC/2021/MAR/238(Q).

Clinical parameters evaluated were PD, measured from the marginal gingiva to the base of the pocket; CAL, which refers to the addition of gingival recession and pocket depth; oral hygiene index-simplified (OHI-S), according to John C. Greene and Jack R. Vermillion in 1964; and sulcus bleeding index (SBI), according to Muhlemann and sons in 1971. For calculating PD and CAL, the occlusal stent was prepared to standardize the measurement. The UNC-15 probe was used to measure PD and CAL.

Informed patient consent was obtained from each patient before the commencement of the study. The study was performed in the Department of Periodontology at Maitri College of Dentistry and Research Center, Anjora, Durg. Before the start of the treatment, all the clinical parameters were evaluated for baseline data. After evaluation, each patient in all four groups underwent SRP.

The control group (Group A) patients who received SRP were instructed on oral hygiene measures and were called after one, three, and six months for follow-up. No oral supplements were prescribed for Group A. Test group patients (Groups B, C, and D) after receiving SRP were instructed on oral hygiene measures and prescribed oral supplements of antioxidant diet pills once a day for 30 days. Test group patients were called for follow-up after one, three, and six months.

Statistical analysis

Statistical analyses of every group were conducted at baseline and six months after the follow-up in IBM SPSS statistics software version 19 (IBM Corp., Armonk, NY). An ANOVA test was performed for intergroup comparison on PD, CAL, and OHI-S. The results showed that these variables were statistically non-significant at baseline and statistically significant following the investigation. Pairwise comparison (intragroup) was carried out for PD and CAL using Tukey's post hoc test. Bleeding on probing analysis was assessed using the Kruskal-Wallis test. P-values of p<0.05 and p<0.001 are considered to be significant.

## Results

On intergroup comparison, there was a significant statistical difference between baseline and post-six-month follow-up for PD. On intragroup comparison for PD reduction, Group D showed a reduction in PD, followed by Group B, which was non-significant with Group D, followed by Group C, and Group A, which was enormously significant with Group D after six months of follow-up (Group D > Group B > Group C > Group A). On intergroup comparison, there was a statistically non-significant variation between baseline and post-six-month follow-up for OHI-S. On intergroup comparison, there was a significant statistical difference between baseline and post-six-month follow-up for CAL (Tables 1-2).

**Table 1:** Mean and standard deviation of pocket depth (reduction) in mm after using all of the treatment modalities after applying the ANOVA test A p-value of p<0.000 was considered highly significant.

Study groups	Pocket depth in mm (Mean ± S.D.)	F-value	p-value
Group A (Scaling and root planning)	1.55± 0.60	36.16	0.00000(P<0.000)
Group B (Lycopene)	3.1±0.78		Highly significant
Group C (Septilin)	2.25±0.44		
Group D (Green coffee beans)	3.35 ±0.58		

**Table 2:** Multiple pairwise comparisons among different groups with respect to pocket depth (in mm) after applying Tukey’s post hoc test P-values of p<0.05 and p<0.001 are considered to be significant.

Groups Comparison		Mean±S.D.	p-value
				Group A (Scaling and root planning)	Group B	3.1±0.78	0.001
Group C	2.25±0.44	0.0029		
Group D	3.35 ±0.58	0.001		
Group B (Lycopene)	Group A	1.55± 0.60	0.001
	Group C	2.25±0.44	0.0002
	Group D	3.35 ±0.58	0.572
Group C (Septilin)	Group A	1.55± 0.60	0.0029
	Group B	3.1±0.78	0.0002
	Group D	3.35 ±0.58	0.001
Group D (Green coffee beans)	Group A	1.55± 0.60	0.001
	Group B	3.1±0.78	0.572
	Group C	2.25±0.44	0.001

On intragroup comparison for CAL reduction, Group D showed a reduction in CAL, which was followed by Group B, which was non-significant with Group D, followed by Group C, which was significant with Group D, followed by Group A, which was highly significant with Group D after six months. On intergroup comparison, there is no statistically significant variation between baseline and post-six-month follow-up for bleeding on probing as per index (Tables 3-4).

**Table 3:** Mean and standard deviation of reduction in clinical attachment loss in mm after using all of the treatment modalities after applying the ANOVA test A p-value of p<0.000 was considered highly significant

Study groups	Clinical attachment loss in mm (Mean ± S.D.)	F-value	p-value
Group A (Scaling and root planning)	2.00± 0.85	36.15	0.00000 (p<0.000)
Group B (Lycopene)	3.85±0.58		Very significant
Group C (Septilin)	3.2±0.41		
Group D (Green coffee beans)	3.9±0.71		

**Table 4:** Multiple pairwise comparisons among different groups with respect to clinical attachment level (in mm) after applying Tukey’s post hoc test P-values of p<0.05 and p<0.001 are considered to be significant.

Groups comparison		Mean±S.D.	P-value
				Group A (Scaling and root planning)	Group B	3.85±0.58	0.001
Group C	3.2±0.41	0.001		
Group D	3.9±0.71	0.001		
Group B (Lycopene)	Group A	2.00± 0.85	0.001
	Group C	3.2±0.41	0.0132
	Group D	3.9±0.71	0.9951
Group C (Septilin)	Group A	2.00± 0.85	0.001
	Group B	3.85±0.58	0.0132
	Group D	3.9±0.71	0.0065
Group D (Green coffee beans)	Group A	2.00± 0.85	0.0001
	Group B	3.85±0.58	0.9951
	Group C	3.2±0.41	0.0065

On intergroup comparison, there was no significant statistical difference between baseline and post-six-month follow-up for the OHI-S. However, Group C showed better improvement than Group A, followed by Group B, which was followed by Group D (Table 5).

**Table 5:** Mean and standard deviation of the oral hygiene index-simplified (OHI-S) score after using all of the treatment modalities after applying the ANOVA test A p-value of p>0.05 was considered not significant

Study groups	OHI-S score (Mean ± S.D.)	F-value	p-value
Group A (Scaling and root planning)	1.59± 0.59	0.038	0.990(P>0.05)
Group B (Lycopene)	1.58±0.55		Not Significant
Group C (Septilin)	1.61±0.58		
Group D (Green coffee beans)	1.55±0.57		

On intergroup comparison, there is no significant statistical difference between baseline and post-six-month follow-up for bleeding on the probing index. However, Group D showed better improvement than Group C, followed by Group A, which is followed by Group B (Table 6).

**Table 6:** Comparison of the bleeding on probing score (as per index) among all the study groups after using all of the treatment modalities after applying the Kruskal-Wallis test A p-value of p>0.05 was considered not significant.

Study groups	N	Mean rank	Chi-square value	p-value
Group A (Scaling and root planning)	20	41.18	0.428	0.934 (P>0.05)
Group B (Lycopene)	20	37.82		Not significant
Group C (Septilin)	20	41.2		
Group D (Green coffee beans)	20	41.8		

## Discussion

Antioxidants are compounds that, when viewed alongside the concentrations of an oxidizable substrate, will substantially slow down or prevent the substrate from oxidation [3]. It is believed that antioxidant therapy is effective in treating periodontitis [8], reduces oxidative stress, and has a beneficial effect on the tissue, which encourages healing and repairs the tissue's architecture [9]. Therapy with medications that inhibit the manufacturing of free radicals (ROS) or limit their effects may be beneficial in treating tissue destruction, and regulating free radical synthesis appears to be essential for its inhibition [10].

Lycopene is one of the main antioxidants in the diet. It interferes with other non-oxidizing methods, such as anti-inflammatory medicines, and has a strong capacity to scavenge radicals [6]. As the most potent biological antioxidizing agent, lycopene has an unusual ability to bind molecules that react with oxygen [10]. Lycopene is the most robust biological carotenoid and effective natural antioxidant, having a physical settling rate with singlet oxygen that is over 10 times greater than alpha-tocopherol and twice as high as beta-carotene [1]. Belludi et al. have reported that when used in conjunction with full-mouth SRP therapy for the oral cavity, lycopene shows promise as a treatment option for patients with moderate periodontal diseases [10].

Burdock leaves have ample CGA. It has been found that CGA can inhibit periodontitis-related bone resorption [11]. Chlorogenic acid prevents the growth of bacterial pathogens by enhancing the permeability of the outer membrane, which in turn causes potassium to move out of the cell, eventually resulting in a rupture in the membrane and allowing the cytoplasmic contents, including nucleotides, to escape [12]. In their study, Tsou et al. found that CGA has a dose-dependent prohibitive effect on protease activity in *P. gingivalis*. Strains of *P. gingivalis*, when subjected to CGA concentrations at a minimum bactericidal concentration (MBC) and 1/2 × MBC, showed a drop in protease activity of more than 70% compared to the control group [13, 14].

In the current research, the green coffee beans group resulted in a reduction in PD and CAL, which is statistically significant compared to the Septilin and SRP groups. The active component in unroasted green coffee beans that gives them their antibacterial qualities is called CGA. The antioxidant properties of this extract are linked to the presence of polyphenols, which are strong antioxidants that can neutralize free radicals by donating an electron or hydrogen atom [15]. The current research findings are dependable with those of Bharath et al., who, in their in vitro study, concluded that green coffee beans were effective against four periodontopathogenic bacteria at low concentrations. 0.2 μg/ml of concentration is the minimum inhibitory concentration of *P. gingivalis*, *P. intermedia*, and *A. actinomycetemcomitans*, whereas *F. nucleatum* is resistant at a concentration of 1.50 μg/ml but demonstrated sensitivity at 3.125 μg/ml. Thus, he concluded that the antimicrobial activity of green coffee beans as an adjunct to SRP is effective in opposing *P. gingivalis*, *P. intermedia*, *F. nucleatum*, and *A. actinomycetemcomitans* in the management of PD [4].

On comparing the results of the green coffee beans and lycopene groups, the CGA group showed better results in the reduction of PD and CAL but was not statistically significant. Antioxidant supplementation has been shown to attenuate endogenous antioxidant depletion, thus alleviating associated oxidative damage in various clinical studies. To the best of our knowledge, there is no comparable study evaluating CGA and lycopene. Rather, a study done by Tripathi P et al. compared full-mouth oral prophylaxis with lycopene with green tea extract, and results concluded that the combination showed highly significant improvement in plaque index and SBI [1], which is comparable to the results of the present study, where there is significant improvement from baseline but is not statistically significant on intergroup comparison of SBI and OHI-S index. On intergroup comparison, Septilin showed better CAL, PD, SBI, and OHI-S reduction than SRP alone, which is comparable with the study of Deore et al., where results suggested a significant reduction in CAL and SBI in the Septilin group than only the SRP group [3]. To date, there is no literature available to compare green coffee beans, lycopene, Septilin, and SRP in stage III grade B periodontitis.

Taking into account the study's limitations, which include its small sample size and short follow-up period, larger sample sizes and additional research are needed to validate the findings.

## Conclusions

The idea of ROS-induced degradation has prompted researchers to look for a suitable antioxidant therapy to complement existing treatments for a variety of illnesses in the category of inflammatory periodontal diseases. Antioxidant oral supplements can be recommended to all patients as they are beneficial for oral as well as general health. In the current study, green coffee bean extract showed significant improvement over Septilin and SRP, but it did not show statistically significant improvement over lycopene. Green coffee bean extract and lycopene may prove to be an encouraging adjunctive therapeutic and prophylactic fashion along with SRP in the treatment of stage III grade B periodontitis.
